# Application of Collagen I and IV in Bioengineering Transparent Ocular Tissues

**DOI:** 10.3389/fsurg.2021.639500

**Published:** 2021-08-26

**Authors:** Yihui Song, Morgan Overmass, Jiawen Fan, Chris Hodge, Gerard Sutton, Frank J. Lovicu, Jingjing You

**Affiliations:** ^1^Save Sight Institute, Faculty of Medicine and Health, The University of Sydney, Sydney, NSW, Australia; ^2^Key Laboratory of Myopia of State Health Ministry, Department of Ophthalmology and Vision Sciences, Eye and Ear, Nose, and Throat (ENT) Hospital, Shanghai Medical College, Fudan University, Shanghai, China; ^3^New South Wales (NSW) Tissue Bank, Sydney, NSW, Australia; ^4^Vision Eye Institute, Chatswood, NSW, Australia; ^5^Discipline of Anatomy and Histology, School of Medical Sciences, The University of Sydney, Sydney, NSW, Australia; ^6^School of Optometry and Vision Science, University of New South Wales, Sydney, NSW, Australia

**Keywords:** bioengineering, collagen type IV, cornea, lens, retina, collagen type I

## Abstract

Collagens represent a major group of structural proteins expressed in different tissues and display distinct and variable properties. Whilst collagens are non-transparent in the skin, they confer transparency in the cornea and crystalline lens of the eye. There are 28 types of collagen that all share a common triple helix structure yet differ in the composition of their α-chains leading to their different properties. The different organization of collagen fibers also contributes to the variable tissue morphology. The important ability of collagen to form different tissues has led to the exploration and application of collagen as a biomaterial. Collagen type I (Col-I) and collagen type IV (Col-IV) are the two primary collagens found in corneal and lens tissues. Both collagens provide structure and transparency, essential for a clear vision. This review explores the application of these two collagen types as novel biomaterials in bioengineering unique tissue that could be used to treat a variety of ocular diseases leading to blindness.

## Introduction

The cornea and lens facilitate a pathway for light to pass through the eye to reach the retina, which then receives and transfers visual signals onto the brain for processing. These three major ocular tissues are critical for generating clear vision; therefore, any damage to the cornea, lens, and/or retina will undoubtedly impair eyesight and often lead to blindness. Tissue engineering, in particular, the development of biomaterials with specific properties, has been increasingly researched for treating ocular disease (1). Due to its abundance in the corneal stroma, collagen type I (Col-I) has been a popular and versatile biomaterial developed to replace diseased corneal layers; however, it has become evident that no singular biomaterial can be an effective substitute for the intact cornea because of the differences in composition between each of the corneal layers (2). Collagen type IV (Col-IV) is the predominant member of Descemet's membrane of the cornea, the supportive layer of the corneal endothelium (3, 4). Furthermore, it is also the main collagen type detected in the lens capsule (5), and in both Bruch's membrane and the internal limiting membrane (ILM) of the retina (6). Therefore, the application of Col-IV as a biomaterial could potentially be useful in creating a natural environment and substratum for corneal endothelial cells and the epithelial cells of the lens and retina. In this paper, previous publications on Col-I and -IV in ocular-related applications were reviewed and the insights into the future direction of development of these two collagen types in ocular bioengineering are discussed.

### The Distribution of Col- I and Col-IV in the Cornea, the Lens, and the Retina

**Figure 1:**
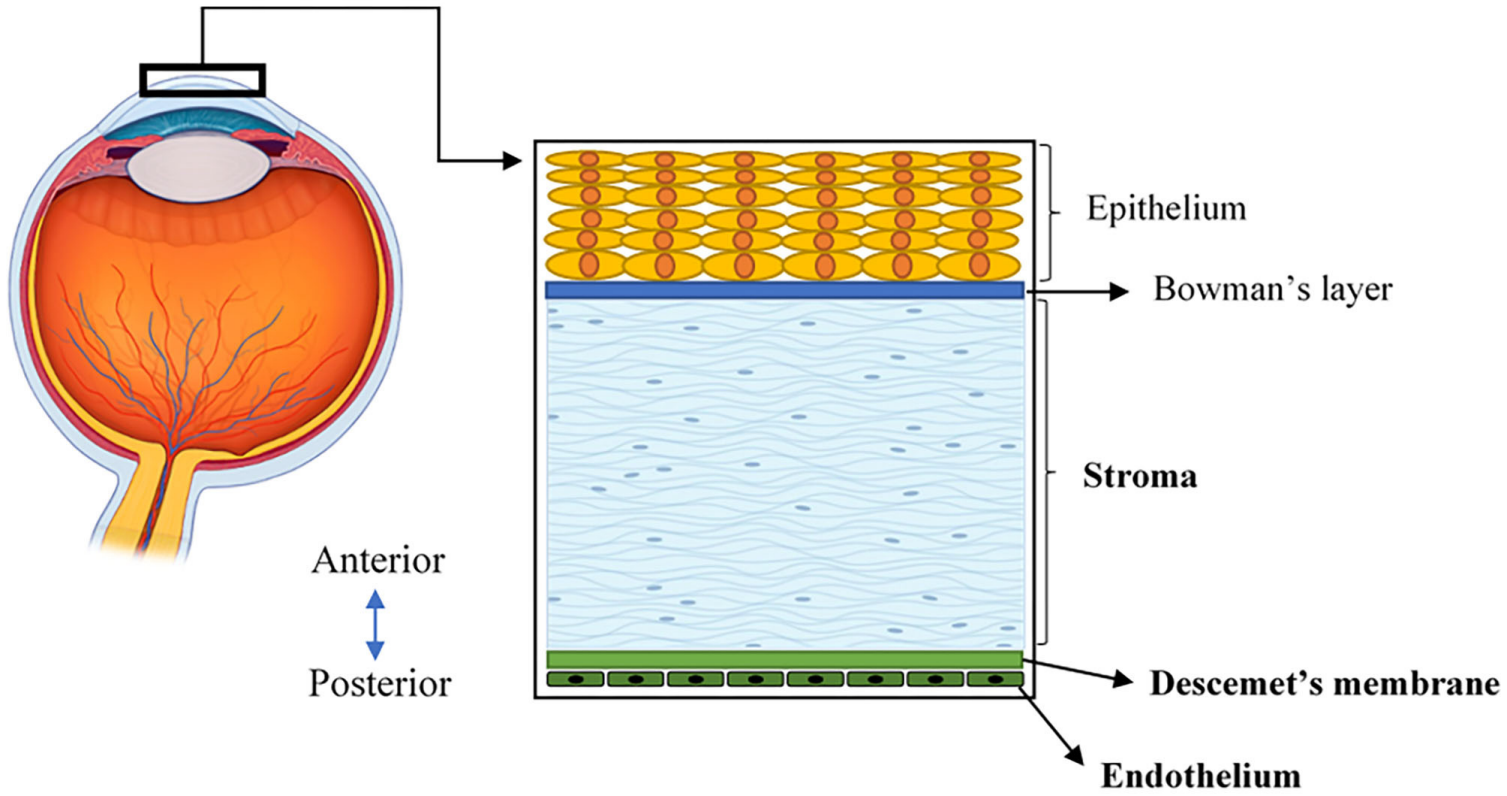
A schematic illustration of the human cornea, located at the front of the eye and consisting of five layers.

The human cornea is a transparent, avascular, highly innervated, and organized tissue that is located at the front of the eye (Figure 1). The cornea acts as a transparent window making up two-thirds of the refractive power of the eye and consists of five main layers from anterior to posterior sides: corneal epithelium, Bowman's layer, corneal stroma, Descemet's membrane, and corneal endothelium (2) (Figure 1). One major structural protein in the cornea is collagen. The human cornea consists of many types of collagen and different collagen combinations are detected within different layers (Table 1).

**Table 1:** Distribution of collagen types in the human cornea.

**Layer**	**Collagen type**
Epithelium	IV, XV, XVIII, XII (7–13)
Bowman's layer	I, III, V (14)
Stroma	I, III, V, VI, XII, XIV (15–17)
Descemet membrane	IV, VIII (15, 18)

The central thickness of a normal adult human cornea approximately measures 530 μm (19). In the context of bioengineering, the stroma is critical because it constitutes the majority of the corneal volume and provides a significant contribution to both its overall transparency and strength (20). The corneal stroma is predominantly made up of Col-I fibrils organized into ~300 orthogonally arranged lamellae (2). These fibrils have a unique, smaller diameter, and regular interfibrillar spacing that supports the transparency of the tissue (21). Furthermore, when stress is applied to the cornea, these well-organized collagen fibers of the stroma are stretched to counterbalance this force, allowing the cornea to maintain its existing shape (22). In addition to the stroma, the corneal endothelium is comprised of a monolayer of interconnected hexagonal cells sitting on the Descemet's membrane. This layer is key to maintaining relative stromal deturgescence/dehydration that is essential for corneal transparency (2). The Descemet's basement membrane is comprised primarily of Col-IV, as well as laminin, perlecan (a heparan sulfate proteoglycan), nidogen, and to a lesser degree, collagen type VIII (Col-VIII) (3, 4). While both Col-IV and Col-VIII are present in the Descemet's membrane, only Col-IV is located adjacent to endothelial cells in both the infant and adult structure (19). In comparison, Col-VIII chains initially face the endothelial cells in the infant Descemet's membrane, but lose contact as we age and shift to face the stroma (3, 4).

**Figure 2:**
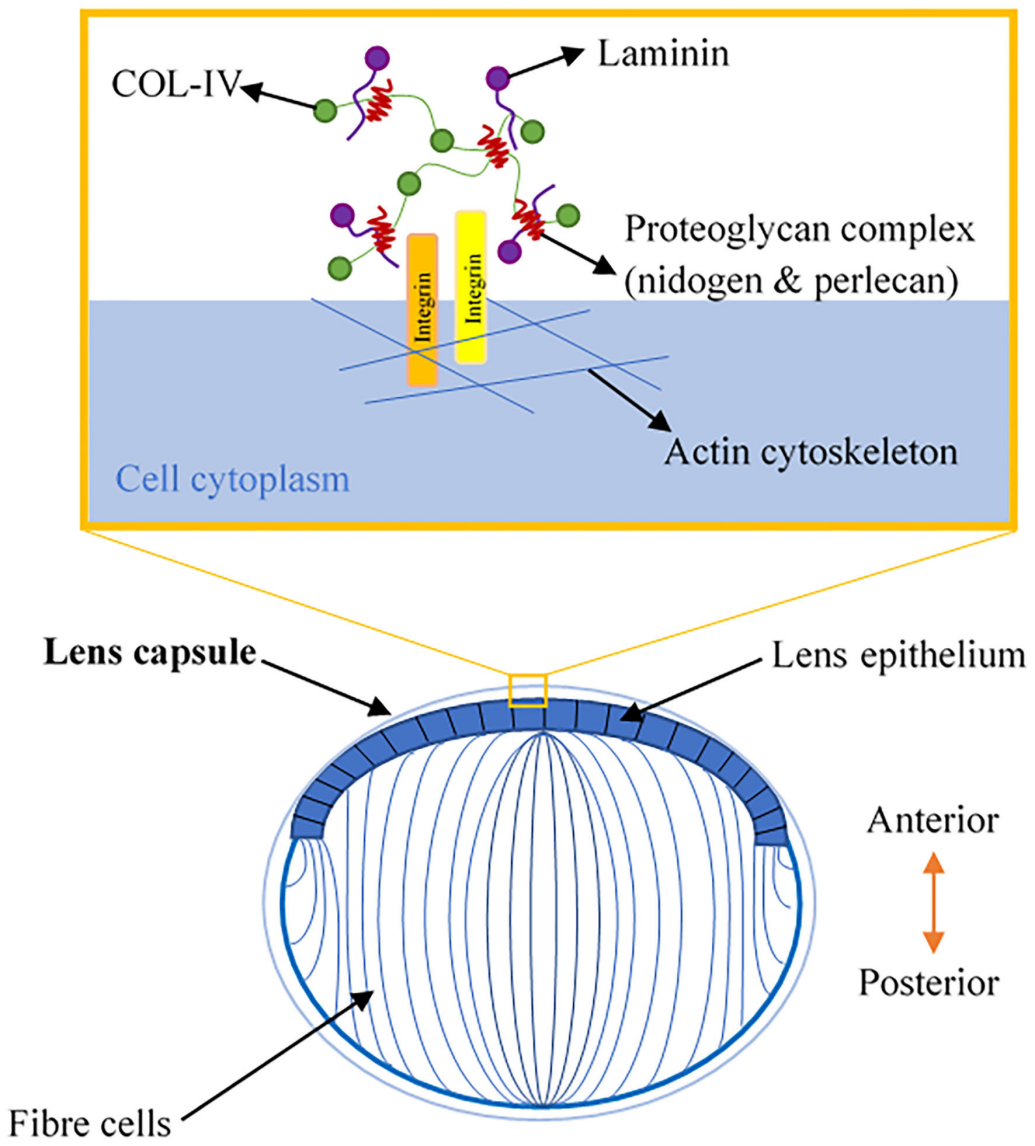
A schematic illustration of lens anatomy and the matrix composition of the lens capsule in the enlarged area.

The lens is a transparent, biconvex orb that consists of the lens capsule, the lens epithelium, and lens fibers (Figure 2). Col-IV is the main type of collagen found in the lens capsule, which is a thick, uninterrupted basement membrane surrounding the lens. The lens capsule is structurally analogous to the corneal Descemet's membrane, as it consists of interlinking Col-IV and laminin networks bound together by nidogen and perlecan (5) (Figure 2). The lens capsule acts as a supporting matrix for lens epithelial cells anteriorly and fiber cells posteriorly. As a result of this structure encapsulating all lens cells, it also protects them from infection. In younger eyes, the lens capsule also has a role in determining the force required for lens accommodation, a process where the lens changes shape to alter our field of focus (23).

**Figure 3:**
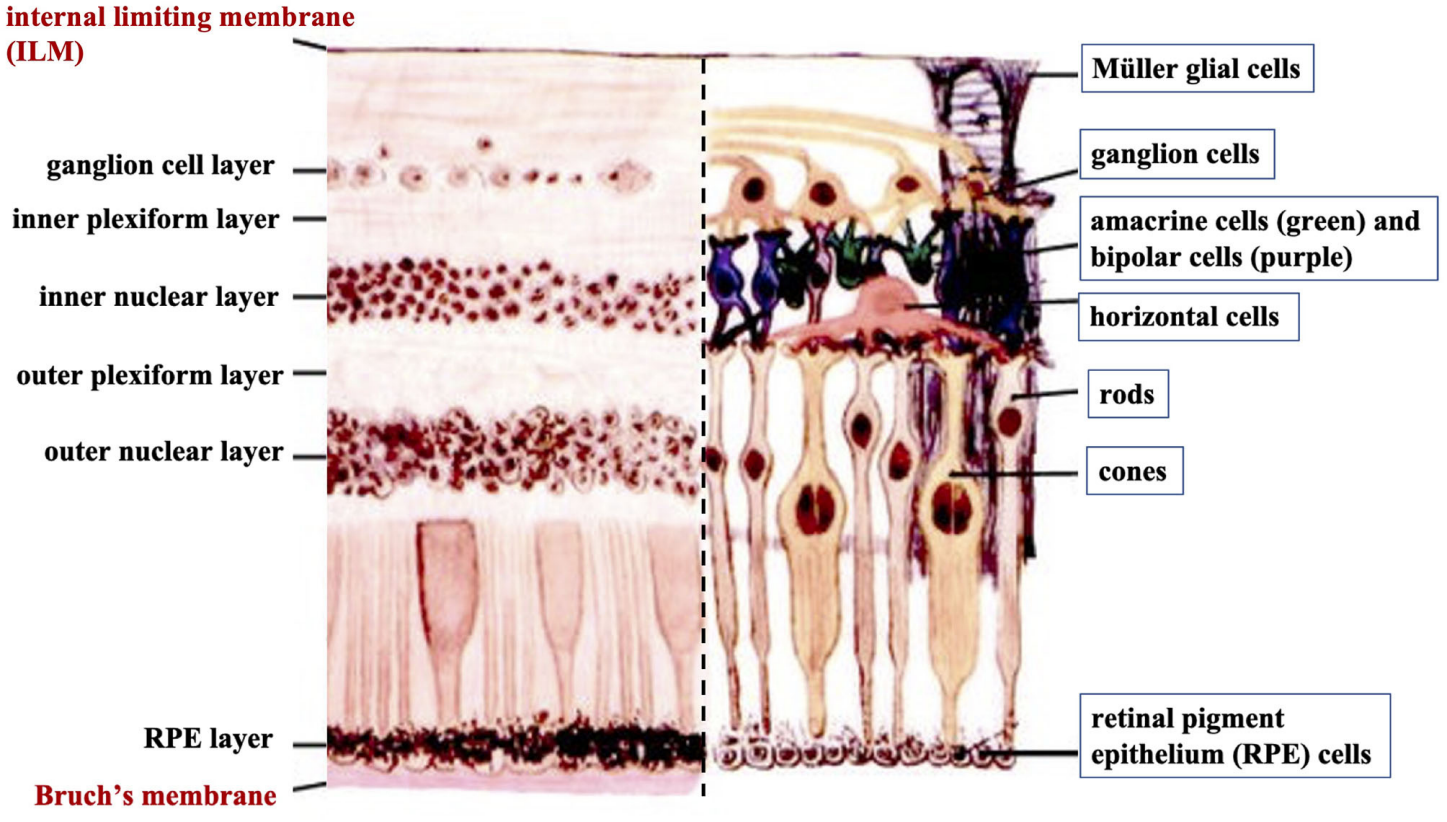
A cross-sectional histological image of the retina, with its different layers (left) and the corresponding diagrammatic image depicting the different cell types of the retinal neural layers (right).

The human retina is the sensory tissue that lines the inner surface of the back of the eye, which senses light and sends signals to the brain to provide vision (6). It contains multiple layers with various cell types (Figure 3). Retinal ganglion cells (RGC) represent a type of neuron located at the inner surface of the retina. The RGCs receive visual information from the photoreceptors (rods and cones) *via* intermediate neuron types including bipolar cells, amacrine cells, and horizontal cells. Rods and cones are responsible for sensing light. Bipolar cells transfer visual information from photoreceptor cells to amacrine cells. Amacrine cells are interneurons in the retina that have short neurotic processes to connect to adjacent neurons and to transfer neuronal signals. Horizontal cells, which are the laterally interconnecting neurons, have cell bodies in the inner nuclear layer of the retina. They help integrate and regulate the input from multiple photoreceptors (6). Retinal pigmented epithelial (RPE) cells make up a single layer of the postmitotic cells. This epithelia functions as a natural barrier and a regulator of the overlying photoreceptors (24). The final type of retinal-specific cells is the Müller glial cells. These cells span the entire retina and connect with all other cell types *via* cellular processes that reach out to wrap around the neurons and the synapses. They also reach out to blood vessels, so as to act as an intermediary between neurons and the circulatory system, thus regulating the flow of nutrients to the retina. Müller glia plays a critical role in maintaining neuronal health and supporting visual function (6). When light first enters the retina, it passes through the ganglion cell layer (GCL), then the inner plexiform layer (IPL), inner nuclear layer (INL), outer plexiform layer (OPL), and outer nuclear layer (ONL) (6). All these neural layers comprising eight types of retinal cells are located between the ILM and Bruch's membrane (Figure 3).

Basement membranes are specialized structures of the extracellular matrix that play an essential role in tissue development and maintenance. Type IV collagens are abundant components of all basement membranes (25). Bruch's membrane and ILM represent two significant basement membranes within the human retina and are located at the inner and outer retina, respectively (Figure 3) (6). Bruch's membrane primarily regulates the passage of nutrients and metabolites between the RPE and underlying choriocapillaris (6). Bruch's membrane also offers a solid base and attachment site for RPE cells, acting as a part of the blood-retinal barrier (26). Bruch's membrane may also be involved in RPE differentiation (27) and wound healing (28, 29). Type IV collagen is present on both sides of Bruch's membrane in a sandwich style with the middle layer containing elastic fiber-like bands, and can also be detected in the extracellular matrix surrounding human RPE cells (30).

The ILM is not a true membrane, resulting from the fusion of the foot processes of the glia-like Müller cells. It forms a physical barrier that protects the retina from toxins and from traction from the vitreous as the eye moves. Col-IV is the predominant extracellular matrix (ECM) protein in human ILM and accounts for ~60% of its total proteins (31). Col-IV has been detected throughout the entire thickness of ILM and is likely to be secreted by retinal Müller cells (32). Several studies have identified that Col-IV is critical not only for the structural integrity of the basement membrane but also for neuron survival and angiogenesis (33, 34). Higher expression of Col-VI has been found on the posterior side of the retina compared to its anterior side (35).

### The Structure of Col-I and -IV

Collagens make up a supra-family of ECM proteins possessing a distinct triple-helical region formed from three polypeptide chains (36). Currently, 28 genetically distinct collagen types have been identified and described in the literature (37, 38). Within this group, Col-I is classified as fibril-forming, while Col-IV is defined as network-forming due to their unique supramolecular organization. Due to its predominance in body tissues, Col-I biosynthesis has been more extensively explored and will be outlined in this review; however, there is a notable lack of focus on the differences between Col-I and Col-IV biosynthesis, which can be predicted on the basis of their differing supramolecular structures.

At its most basic level, collagen biosynthesis involves the processing and aggregation of collagen monomers into functional structures. Synthesis begins within the nucleus in generating relevant mRNAs, followed by the transcription of mRNA molecules into a different α-chain (38). While Col-I only possesses two types of α-chains (α1 and α2), Col-IV has 6 α-chains (α1–6) that form different network configurations to provide basement membrane specificity (39, 40). Col-I and Col-IV are also considered heterotrimeric. This classification results from the number of α-chain genes associated with each subtype; for example, Col-I trimers are composed of two α1 chains and one α2 chain (39). Col-IV heterotrimers have greater complexity as they can organize into three different isoforms: α1α1α2, α3α4α5, and α5α5α6. The α1α1α2 Col-IV heterotrimer is predominant throughout the basement membranes of the body (40); however, the α3α4α5 network has been identified within specific tissues, including basement membranes within the eye. The adult human lens capsule contains only α3α4α5 network (41), whereas only α1α1α2 was found in the retinal ILM (42). Both α1α1α2 and α3α4α5 collagen IV networks co-exist in Bruch's membrane (43), and all of six isoforms have been detected in adult Descemet's membrane (3).

Within the primary structure of collagen is a high proportion of the repeating triplet sequence, Gly-X-Y. X, and Y in this sequence are predominantly occupied by proline (Pro) and hydroxyproline (Hyp), respectively (38). High proportions of Hyp are essential as this amino acid has a critical role in stabilizing the triple helix through the formation of intramolecular hydrogen bonds. Accordingly, Xu et al. (2019) found hydrogen bond energy within helical regions to positively correlate with greater thermal stability (44). The formation of the triple helix in the procollagen molecule is likely a shared process for Col-I and Col-IV. While uninterrupted triple helical domains are the dominant structure of Col-I and have been found to have a defined length of 300 nm (45, 46), Col-IV instead contains 21–26 interruptions within the Gly-X-Y sequence of the triple helix, leading to greater intramolecular flexibility, more suited for network formation (47, 48).

Within the extracellular space, self-assembly is initiated, and due to the significant differences in the resulting matrices, Col-I and Col-IV deviate at this stage. Released Col-I tropocollagen molecules undergo a spontaneous but organized aggregation process (38); albeit this spontaneous process has also been found to be dependent on temperature, pH, ionic strength of the solution, and the concentration of collagenous and non-collagenous components (38, 49). Molecular assembly of Col-I involves a linear alignment, with N- and C-terminal ends opposed in different tropocollagen trimers. Formed elongated fibrils can be 500 μm or more in length with a width of 500 nm (36, 38). These fibrils also have a specific 3-dimensional packing arrangement involving lateral associations between fibrils as they are staggered by about one-fourth of a molecular length. This staggering also provides Col-I fibrils with a striated organization where bands appear every 67 nm (50). Fibrillar organizations of Col-I show a degree of crystallinity; however, this organization varies throughout different tissues. Col-I aligns into straight parallel fibrillar arrangements in tendons, while in the human corneal stroma, Col-I fibrils are arranged in 300 orthogonally arranged sheets (51, 52).

Following the spontaneous molecular arrangement, additional stabilization of collagen is provided through crosslinking. Lysyl oxidase (LO) facilitates crosslink formation both in the head-to-tail alignment between adjacent telopeptide regions and in adjacent helical regions laterally (53, 54). LO initiates the formation of aldehydes from previously modified amino acid residues, lysine, and hydroxylysine (38). Aldehydes formed in each region are then able to trigger aldol reactions with lysine residues in adjacent molecules, resulting in the formation of aldimine crosslinks. Intermolecular crosslinks following spontaneous molecular organization provide the required mechanical strength and stability for collagen organization. In comparison, Col-IV supramolecular assembly aims to create a mesh-like network structure. Within its polypeptide structure, Col-IV chains have an N-terminal collagenous 7S domain, and a C-terminal non-collagenous/globular domain (NC1), in addition to the central triple helix (40). In the creation of a network, varying arrangements of Col-IV trimers are created. Two Col-IV trimers can covalently interact *via* their NC1 domain to form dimers while four 7S domains are able to crosslink into tetramers allowing for the creation of a strong and stable network (40, 47, 55). More specifically, the 7S domains bond through the formation of disulphide bridges and covalent bonding of lysine and hydroxylysine residues (47). This is a unique feature seen in the Col-IV quaternary structure as the 7S domain contains cysteine and lysine residues. LO also plays a role in Col-IV crosslink formation as it again facilitates oxidative deamination of lysine and hydroxylysine allowing for the formation of aldimine links, as seen in Col-I (56).

## Current Development of Tissue Engineering in Treating Ocular Diseases

The ability to manufacture bioengineered tissue that mimics existing intact tissue that can act as a means of repairing damage or to replace diseased layers presents obvious benefits in disease treatments. Using native matrix protein as the base material is a plausible direction and collagen, in particular Col-I, has been widely investigated as a suitable candidate biomaterial. The current landscape of tissue engineering in cornea, lens, and retina is detailed in Section Cornea.

### Cornea

Corneal blindness is a worldwide problem that affects at least 10 million people (57–59). Corneal transplantation is an effective way to treat corneal blindness; however, there are still several significant barriers to this procedure including shortage of donor tissue and graft rejection. Currently, only one cornea is available for every 70 patients worldwide (60). The lack of fully functional eye bank facilities in third world countries, usually accompanied by other limitations, such as the lack of staff training, equipment, and public awareness of corneal donation, impact the access and ability to complete this vision rehabilitative procedure (61). High tissue graft rejection rates have also been reported potentially leading to reduced visual acuity. One study found that 10% of grafts are rejected within the 1st year, increasing in up to 50% of patients who have had multiple prior graft procedures. Each rejection episode represents a risk of total graft failure and permanent blindness (62). Donor viability may be further impacted by the presence of transmissible diseases like hepatitis A and HIV. Similarly, donor numbers may be impacted by the increasing popularity of corneal laser refractive surgery which represents a relative contraindication for use in corneal transplant procedures (58).

Corneal tissue engineering has become increasingly popular to treat severe corneal injuries and is used in two main applications: constructing a bioengineered tissue to replace donor tissue and serving as a filler/implant to fill/replace partial damaged tissue. Due to its abundance within the cornea, Col-I is one of the main natural polymers studied in this area. Researchers have developed and used plastically compressed collagen (PC) to construct bioengineered corneal grafts (63). PC refers to collagen gel from rat-tail collagen I being self-crosslinked at 37°C, and then compressed and dehydrated to provide strength and corneal shape. During this process, keratocytes (corneal cells located in the stroma) may be seeded into the structure. It is reported that minimal cell death was induced by the compression of the gel, where the collagen fibers were dense and homogeneous, similar to that of the intact corneal stroma (64). Its strength and optical properties can be further improved by introducing electrospun poly(lactic-co-glycolic acid) (PLGA) mats and using a laser to create micro-holes in the matrix resulting in increased (15 times higher) light transmittance than the previous model (65).

There are other physical methods to make collagen-based corneal implants, including centrifugal ultrafiltration and vitrification (66, 67). The process of centrifugal ultrafiltration involves the concentration of a collagen solution (from 5 to 125 mg/ml) with 30 h of centrifugation, followed by rehydration in water with an additional 10 h of centrifugation. The final collagen solution was neutralized and molded into a corneal shape. The Young's modulus of the structure was 4.83 MPa, which was between the strength of anterior corneal stroma (9.72 MPa) and posterior corneal stroma (2.04 MPa) (66). Further crosslinking including photocrosslinking or chemical crosslinking were also tested, displaying a mean light transmittance rate of over 85% and higher Young's modulus (34.89 MPa), compared to the native corneal stroma (66). This was also compatible with keratocytes and corneal epithelial cells, further representing the ability to closely mimic natural tissue (66). Collagen structures produced by vitrification, called “Collagen vitrigel,” can support re-epithelialisation on its surface, and stop epithelial cells from migrating into the cornea stroma (68). An attempt to increase its optical and mechanical properties was achieved by mixing the collagen solution with β-cyclodextrin to regulate collagen fibers to better mimic the normal corneal stromal structure (69). This resulted in comparable mechanical properties to the native cornea; however, only with moderate transparency (60–80% light transmission in visible light range), representing a relative disadvantage of this process. It was also found that the optical properties of the collagen vitrigel can be improved by replacing β-cyclodextrin with α-cyclodextrin, and inducing further chemical crosslinking with 1-ethyl-3-(3-dimethylaminopropyl)carbodiimide hydrochloride (EDC), suggesting further improvements may still be possible (70).

Chemical crosslinking is widely used in fabricating bioengineered corneal implants. The most common chemical crosslinker is EDC and N-hydroxysuccinimide (NHS). Animal collagen (porcine collagen) and recombinant human collagen (type I and type III) have been crosslinked with EDC and NHS to fabricate an artificial cornea (71–73). These collagen structures have shown good mechanical properties (with up to 260 KPa tensile strength), optical properties (with up to 92.5% light transmittance), and show compatibility with corneal cells (71, 73). Other materials, such as silk fibroin, may be added into the chemically crosslinked collagen hydrogel prior to crosslinking, to enhance certain mechanical properties, such as maximum tensile strain without affecting the biocompatibility (74).

Electro-compacted (EC) collagen gels are produced by compacting collagen using a pH gradient created by electrodes. This can improve the packing density of the collagen gel. Kishore et al. developed a collagen matrix using this method (75). The collagen matrix was further crosslinked by EDC and NHS to enhance its strength. The results show that although chemical crosslinking reduced the visible light transmission (from 79–93% to 67–89%), it dramatically increased the tensile modulus of the collagen gel (from 16 kPa to 1.8 MPa) (75). The structure is also shown to be compatible with primary keratocytes. Another bioengineered corneal stroma layer fabricated by electro-compaction and stacking collagen film has been developed by Chen et al. (76). The EC-compacted collagen solution showed a 5-fold of increase of storage modulus compared to non-EC compacted collagen and remains capable of promoting the proliferation of human keratocytes. These collagen layers, with aligned collagen fibers and human corneal stromal cells cultured on them, can be stacked and integrated by weighting down, to form a layered microstructure that closely mimics the corneal stromal structure. No further chemical crosslinking processes are introduced in the weighting down process, with the stacked structure having a much lower Young's modulus than the native cornea (0.23 kPa compared to 23.05 kPa) (76). Alternatively, magnetic fields have been used to align the collagen fibers during the self-assembling process of the collagen gel (77). The magnetic field-generated collagen gel had a similar arrangement of collagen fibers to the EC collagen gel, and it also supported keratocyte growth (77). Proteoglycans extracted from porcine corneal tissues (35% of decorin and 65% of lumican, keratocan, and osteoglycin) have also been incorporated during the fabrication of the collagen gel to improve the transparency; however, the details of the transmittance was not reported (77).

While there are many studies on collagen-based corneal implants in development, the development of collagen-like material-based corneal fillers/sealants are more recent. Collagen-like material-based filler can be used to seal corneal perforations and has been made and tested by Samarawickrama et al. (78). The collagen-like material-based filler was based on a modified collagen peptide conjugated to polyethylene glycol (CLP-PEG). After its application to the wound site, the filler was further crosslinked by 4-(4,6-dimethoxy-1,3,5-triazin-2-yl)-4-methylmorpholinium chloride (DMTMM) to form a structure that adheres to and seals the perforation. DMTMM was tested individually with human epithelial and endothelial cell lines and was shown to have no cell toxicity. The performance of this filler was compared to cyanoacrylate glue, a currently used treatment in clinics, with the results showing that the CLP-PEG filler glue, with an internal collagen patch, generated a much smoother surface than the cyanoacrylate glue. One disadvantage was that the bursting pressure of the CLP-PEG filler was much lower than the cyanoacrylate glue (86.6 mm Hg compared to 325.9). According to Islam et al., the CLP-PEG hydrogel is significantly weaker than normal human cornea due to its higher water content (90% compared to 78%) (79). While *in vivo* safety has been proven after 5-week long animal experiments done by implanting the gel into the cornea of guinea pigs (78), its weaker mechanical properties raise the concern of whether the CLP-PEG hydrogel may be stable under constant internal pressure for an extended period of time. The main advantages and disadvantages of the collagen-crosslinking method in corneal bioengineering are summarized in Table 2.

**Table 2:** Main advantages and disadvantages of Collagen-crosslinking method in corneal bioengineering.

**Collagen-crosslinking method**	**Main advantages in corneal bio engineering**	**Main disadvantages in corneal bio engineering**
Physical-crosslinking	High mechanical strength (65)	Lower optical properties (65, 70)
Chemical-crosslinking	High mechanical strength (71) High optical properties (73)	Potential cell toxicity (80)
Photo-crosslinking	Higher biocompatibility (81)	Slow crosslinking process (66)
Electro-compaction	Organized collagen fiber (76)	Lower mechanical property (76)

In conclusion, collagen corneal implants have already achieved good mechanical properties, optical properties, and biocompatibility; however, there are limitations. The collagen gel without additional crosslinking usually has a mechanical property weaker than the human cornea (66). Some of the chemical crosslinkers, such as EDC, while can achieve good mechanical properties, may exhibit health risks if remaining within the implant structure. The implants mentioned above adopt traditional manufacturing processes, such as casting that lack flexibility compared to modern fabrication processes, such as 3-D printing.

### Lens

Although the cornea represents the primary structure responsible for light refraction, the lens remains important for fine-tuning and precisely focusing the light that passes through it to the retina. A cloudy lens will prevent light transmission and can therefore lead to reduced visual acuity, and if significant, blindness. Cataract, a condition of irreversible clouding of the natural lens, is the leading cause of blindness affecting ~20 million individuals globally (82–84). Cataract surgery is currently the only method for treating cataract, with 28 million operations performed annually (85). Cataract surgery requires the removal of the clouded lens material and insertion of a prosthetic intraocular lens (IOL). IOLs have varied biomaterial composition and design in order to produce the best visual acuity outcomes and prevent surgical complications; the most common of which is posterior capsular opacification (PCO). PCO results from remaining LECs post-surgery that are attached to the damaged anterior lens capsule. These cells can undergo an epithelial-to-mesenchymal transition (EMT) as they migrate to the posterior capsule (86, 87). These trans-differentiated cells are contractile and deposit excessive extracellular matrix, including Col-I and Col-III that are not normally found within the normal adult lens (86, 88). These activities cause lens capsular wrinkling and opacification, correlated with loss of vision. Historically, PCO rates were as high as 20–40% of patients at 2–5 years follow-up after surgery (89), albeit more recent figures suggest a significantly decreased incidence. Neodymium: YAG (Nd:YAG) laser capsulotomy is an effective procedure used within the clinic to treat PCO. It involves the disruption of the central posterior capsule by the laser to clear the visual axis, thereby improving visual acuity (90). It uses a solid-state laser with a wavelength of 1,064 nm that can deliver high energy to ocular tissue resulting in tissue disruption without physically touching the tissue (91). Although successful, Nd:YAG laser treatment is not without risk, with damage to the IOL and retinal detachment noted in some cases (90, 92, 93).

Clinical and laboratory-based studies identified two key factors affecting associated PCO incidence: IOL material and design. Historically, most IOLs have been made up of three standard synthetic materials: polymethyl methacrylate (PMMA), silicone, and acrylic polymers. PMMA was the first IOL material; however, PMMA IOLs were rigid and inflexible requiring a larger incision in surgery for appropriate insertion (>5 mm). This has previously been correlated with an increased risk of PCO due to the disruption of the blood-aqueous barrier and lens capsule (94, 95). In comparison, foldable IOLs (silicone and acrylic polymers) requiring only small incisions (<2.5 mm) are associated with fewer complications resulting in more widespread use (96, 97). There is a strong influence of IOL material on PCO incidence. Past studies have found that PMMA IOLs are consistently associated with high rates of PCO in comparison to silicone or acrylic IOLs (98, 99); however, recent comparative studies have presented mixed results concerning PCO risk in silicone and acrylic IOLs. It has been found that instead of the material itself defining biocompatibility, the material's hydrophobicity/hydrophilicity may be the defining factor for PCO incidence. IOLs with a hydrophobic character produce significantly lower rates of PCO in comparison to hydrophilic IOLs (100). In addition, when comparing PMMA, silicone, hydrophobic acrylic, and hydrophilic acrylic IOLs, the hydrophobic acrylic IOL produced significantly less PCO compared to other materials (101). It is widely accepted that this hydrophobicity increases adhesion to the Col-IV of the lens capsule, and therefore creates closer apposition of the IOL and remaining posterior capsule following surgery (102, 103). This close adherence provides a barrier to the migrating transdifferentiating lens epithelial cells (LECs). In comparison, IOLs with hydrophilic character have PCO rates not dissimilar to PMMA IOLs, as they have been described to promote aberrant LEC proliferation and migration (102, 104).

Extracellular matrix molecules including Col-IV, fibronectin, and laminin have been evaluated as potential adhesive coatings or materials for IOLs and have been found to mimic the effects of hydrophobic IOLs, producing minimal PCO (105–107). Past studies have noted that IOLs, either made from Col-IV or with a Col-IV coating produced significantly less PCO (108). In these studies, Col-IV was found to assist in stabilizing damage to the blood-aqueous barrier and preventing EMT transformations that normally initiate fibrotic PCO. The incorporation of native lens capsules containing ECM molecules, in particular, Col-IV, therefore, appears promising but requires more development and further research.

Another significant factor determining PCO incidence is IOL edge design. This factor holds significance as these edges also have the potential to form a physical barrier to LEC movement. Studies comparing PCO outcomes between hydrophobic acrylic IOLs and silicone IOLs both made with sharp edges found no significant differences after 3 years of observation (109–111). Sharp edges in combination with other IOL design factors, like uninterrupted edges and appropriately angled haptics, have all been found to contribute to the reduction in PCO (112–114).

Presently no treatment, surgical technique, or IOL design/material arising from engineering this artificial lens replacement has been found to eliminate PCO completely. Therefore, moving forward, researchers have begun to look at options to create/regenerate natural lens structures, a process in which tissue engineering may play a significant role. Mammals have been found to possess lens regenerative abilities contingent upon the remaining LECs being relatively undisrupted and on an intact anterior and posterior lens capsule (115). This method of lens regeneration is driven by these LECs and is called “LEC-mediated regeneration.” Studies in both rabbits and macaques found that upon fiber cell mass removal, a whole lens structure was able to regenerate on the remaining lens capsule within 7 weeks and 5 months, respectively (116); however, these regenerated lenses showed irregular fiber cell growth that led to the development of opacities. To address this problem, other studies have inserted tissue-engineered scaffolds following fiber mass removal. For example, Gwon and Gruber utilized a biodegradable hyaluronic acid scaffold and found lenses of greater optical clarity and normal fiber arrangement regenerated (117). It is believed these scaffolds provide the necessary mechanical support to the remaining lens capsule and LECs, mimicking the support previously provided by the natural fiber mass (118). Furthermore, this then encourages normal regeneration as opposed to aberrant proliferation and migration of LECs (i.e., PCO also linked to a sudden disruption of contact inhibition) (119, 120). With positive results for Col-IV-coated IOLs previously observed, Col-IV could also be a suitable scaffold candidate to be incorporated in future tissue engineering-based approaches to address both lens regeneration and PCO concerns. This type of approach has yet to be trialed in humans and there are limiting factors to consider. For example, older patients in whom the majority of cataract surgeries are performed (121), have hard cataracts that may require more significant intraocular surgical manipulation. This can lead to a significant loss of crucial LECs and possible damage to the supporting lens capsule, both of which are essential for LEC-mediated regeneration. Adult lenses also have larger capsules “stretched” from years of continuous lens growth (118). This impacts on the mechanical environment present and makes it unconducive to regeneration. Therefore, tissue-engineered scaffolds should consider these elements that may impact an outcome following scaffold implantation. For example, if Col-IV-based scaffolds were developed, they may require specific dimensions or design to appropriately stretch a “looser” capsule. Col-IV as a biomaterial could also be cast into a “patch” and utilized to substitute the lens capsule lost during surgery and hence minimize LEC disruption to maximize regeneration potential. The use of collagen biomaterials or scaffolds in this field is not widely seen and the previous study on the benefits of a Col-IV-based IOL is outdated (105–108). Therefore, moving forward, a tissue-engineering approach, utilizing collagen-based scaffolds to encourage lens regeneration is a potential and promising path that still requires a significant amount of work.

### Retina

Tissue engineering in the retina has previously been investigated, with studies using a range of materials including decellularised natural tissues, such as amniotic membrane, lens capsule, Bruch's membrane (BM), collagen I as well as synthetic materials (122); however, to date, there are no reports of Col-IV usage in such retinal bioprinting studies. It is unclear why Col-IV was not used as a main biomaterial for retinal tissue engineering. Col-IV is an important protein for ocular health. Alport syndrome is the most typical Col-IV-related pathology in the eyes. In 1990, a role for Col-IV in an inherited genetic disease was subsequently discovered when mutations in Col-IV a5, and later Col-IV a3, and Col-IV a4, were found to underlie X-linked and autosomal recessive forms of Alportsyndrome, respectively (123). Ophthalmologic findings include anterior lenticonus characterized by a thin, fragile lens capsule (124), dot-and-fleck retinopathy (125), and temporal retinal thinning (126).

Despite no reports for collagen IV as a bioink, it has been used in retinal gluing. The aim of gluing is to achieve a strong and immediate adhesion between the retina and retinal pigment epithelium (RPE). In 1989, researchers tried to apply “Matrigel” that contained Col-IV and laminin, to study the effects of successful adhesives on retinal cells *in vitro*, and to investigate the potential biocompatibility of substrates. They found that the “Matrigel” preparation stimulates the proliferation of bovine retinal glial cells around retinal breaks. Pre-treatment with fibronectin supported the growth of retinal cells after sealing (127); however, there are little to no further studies on Col-IV as a retinal sealant, with most studies conducted prior to the 1990's. This may be due to advancements in vitreoretinal surgery to treat retinal diseases. More recently, gluing associated with retinal tissue engineering appears to represent a renewed focus in retinal surgery-related developments (122), with further potential application in clinics. Tyagi and Basu performed glue-assisted retinopexy for rhegmatogenous retinal detachments (GuARD) in patients, which allowed early visual recovery while avoiding the problems of gas or oil tamponade and obviating the need for postoperative positioning that represents a significant practical limitation for patients in the early postoperative period (128). Ophthalmologists also found fibrin glue provided a superior adhesive for sealing retinal breaks, while showing no additional adverse effects in patients (129). With the early successes reported in 1989, Col-IV may be a valuable biomaterial that is to be used in gluing applications in clinical surgery.

## Collagen-I and -IV in Bioprinting Ocular Tissues

Bioprinting belongs to 3D printing and is classified as additive manufacturing. The fundamental mechanism adopted here is by stacking materials layer by layer to form a scaffold/structure based on computational images (130). Compared to classic molding methods, bioprinting has been in the spotlight in recent years, with its advantages and capability to generate customized structures based on recorded images, as well as the reproducibility of cell printing. In recent years, publications about *in situ* printing, directly printing biomaterials/cells to injured sites using hand-held printers to reconstruct the wound and promote healing, gave us a glimpse of what future surgeries may be like (131).

### Bioprinting

Unlike materials used in traditional 3D printing such as plastics, the materials used in bioprinting refer to biomaterials, usually organic materials, such as collagen, gelatine and alginate, or bioink with a cell-laden ability (130). The most frequently used methods in 3D bioprinting are inkjet bioprinting, laser-assisted bioprinting, and extrusion-based bioprinting (Table 3). Additional technologies like vat photopolymerisation may also be used in bioprinting.

**Table 3:** A summary of bioprinting methods.

**Printing methods**			**Advantages**	**Disadvantages**
Inkjet bioprinting	SJI		Fast, cost-friendly	Requires low viscosity material
	DOD	Thermo		
		Piezoelectric		
		Electrostatic		
Laser-assisted bioprinting			Fast, more controllable	Limited printable structure, high cell death
Stereolithography (SLA)			Good cell viability	Selective in the material of the bioink
Extrusion bioprinting			Simple, flexible, and low-cost	Requires shear-thinning material

Inkjet bioprinting, which is similar to the conventional inkjet printer, prints the structure by precisely depositing micro-drops of bioinks to a substrate. The inkjet printing technique can be divided into two categories, continuous inkjet printing (SIJ), which means continuously printing a stream of drops whilst selecting the drops that are needed to be printed to the substrate, and drop on demand inkjet printing (DOD), where the ink drop is only ejected out of the nozzle as needed (132, 133). The DOD technique can be further divided by the method used to form the micro-droplets; thermo-inkjet printing, piezoelectric inkjet printing, and electrostatic inkjet printing (134). The DOD technique has been applied to fabricating a corneal-like structure incorporated with corneal stromal cells, thus achieving good cell viability (up to 7 days) (135). Inkjet bioprinting has been investigated in its potential of fabricating other tissues, including bone and cartilage tissues (136, 137), blood vessels (138), and retinal layers (139). Inkjet printing is fast and cost-effective compared to other bioprinting methods (140) but is limited by the requirement of low-viscosity material to prevent clogging during printing (141).

Laser-assisted bioprinting during the printing process involves a pulse laser that is applied to a laser absorption layer, with bioink-containing cells covering its lower surface not directly exposed to the laser. This causes thermal expansion that ejects micro-droplets of the bioink onto the substrate (142). This method can precisely control the type and density of the cells during printing (143); hence, is often used to manufacture scaffold-free cell structures (144). A human corneal-like stroma with high cell viability has been produced with this technique, using a Col-I based bioink and human stem cells (145). Further developments have improved the strength of the laser-assisted bioprinted structure, as evidenced by this technique that is used to successfully print mesenchymal stromal cells for bone regeneration, with the aid of pre-printed nHA-collagen disks (146). Laser-assisted bioprinting does not carry the risk of blocking the printing nozzles and remains a relatively fast process. The main current limitation of this technique is the high rates of cell death during the printing process that may impact the long-term survival of the tissue (142, 147).

Photopolymerisation or photocrosslinking is a further technique used in 3D bioprinting, known as stereolithography (SLA). For this technology, a laser beam is applied directly to the printing material to initiate photopolymerisation/photocrosslinking in a selected area of bioink and the 3D structure is printed layer-by-layer. The bioink used in this printing process is usually required to be photopolymerisable or to contain a photoinitiator to be able to crosslink. As an example, methacrylate gelatine (GelMa) and eosin-Y combined with visible light are common materials and photoinitiators used for this cell printing process (148). This combination has been successfully used to print human corneal-like stroma and shows good cell compatibility post-printing (149). Lithium phenyl-2,4,6-trimethylbenzoylphosphinate (LAP) is another photoinitiator used with GelMa and UV. Other studies printing different tissue or organs, including artificial cartilage and liver using this same combination, also have good cell viability (150, 151). Stereolithography has been adopted in making artificial blood vessels with a photopolymerisable polyacrylate material (152). As the bioink used in printing is required to be photo-cross linkable, this remains a relative limitation of the technique.

Extrusion bioprinting is the most common bioprinting used in current applications (153). During extrusion printing processes, the shear-thinning bioink is extruded from a syringe by the pressure created by either air, a piston, or screw. The extrusion 3D printer can be a single syringe, a multi-syringe, or joint syringes, with coaxial printing tips to meet different needs. Extrusion printing is widely used in tissue engineering with a variety of bioinks. Multi-syringe extrusion printing has been used in printing human skin with two different layers, both dermis and epidermis (154). An example of coaxial extrusion bioprinting has been published in a study that used alginate as the shell that was immediately crosslinked by the calcium ion that was contained in the mixture of GelMa and calcium chloride during the initial mixing process, binding the GelMa together before further crosslinking (155). Extrusion bioprinting has great potential in surgery. In addition to printing the entire structure, it can also be used in *in situ* printing. O'Connell et al. developed an extrusion printing-based hand-held device called a “biopen” for treating cartilage injuries (131, 156). As a hand-held device, it increases surgical dexterity and portability. It adopts the coaxial extrusion printing mechanism and integrates a UV-curing attachment to solidify the material during, and post-printing. Another device, developed by Hakimi et al., is also a handheld 3D printer based on double syringe extrusion printing (157). Targeting a range of tissues, this dispensing method uses a cartridge that applies a crosslinker on the top of the material while printing (157). In the above-reviewed extrusion methods, a viscous bioink is usually required to maintain the shape of the printed structure during the printing process. For low viscous bioinks, a method called “freeform reversible embedding of suspended hydrogels (FRESH)” has been developed. In this method, the bioink was printed in a supporting material to obtain higher resolution and structural support for printing low-viscosity bioinks that have difficulty maintaining the printed shape during printing. This technique has already been used to print a range of human tissue including artificial human corneal stroma and heart tissues (158, 159).

### Collagen-I Based Bioink

Since Col-I is the major component of the human corneal stroma, most of the bioink under current investigation for use in corneal applications contains this as its major constituent; however, this is usually combined with other materials in order to gain enough printability or postprinting mechanical properties. These bioinks can be printed with the earlier mentioned bioprinting methods.

The bioink used in 3D printing of human cornea using FRESH printing by Isaacson et al. was a combination of up to 8 mg/ml methacrylated bovine Col-I and sodium alginate (158). The authors reported that with increased concentration of collagen in the bioink, and the addition of sodium alginate, the printability and transparency were improved. A formulation of the bioink that contained 2.66 mg/ml Col-I and 2% of sodium alginate was reported as their choice of best overall properties (158). Another example of combining bovine Col-I with sodium alginate to make the collagen-based bioink for corneal bioprinting was developed by Kutlehria et al. (160). In this study, they used the SLA technique to fabricate the supporting structure and then used extrusion printing to print corneal stroma-like tissue onto the structure. The collagen in use was an acid-soluble bovine Col-I. The sodium alginate used acted to assist the solidification of the printed structure with the use of calcium chloride as a crosslinking agent. Gelatine, incorporated with the bioink, was added to enhance its printability. The optimized concentrations of the components of the bioink include 4% gelatin, 3.25% alginate, and 5 mg/ml collagen (160). A similar formulation of bioink was used by Wu et al., who also combined collagen with other natural polymers, including gelatine and sodium alginate (161); however, they used normal extrusion-based bioprinting techniques, and Col-I sourced from rat tail. As extrusion printing requires the bioink to have high printability, this bioink has high gelatine content at 10% weight per volume, to improve printability. The collagen concentration was low at 0.83 mg/ml and the bioink also contained 1% alginate. The printed structure was immersed with calcium chloride to further strengthen the structure. The printed structure was found to be transparent and cell compatible; however, the authors also reported that the alginate structure could not be degraded by cells, therefore potentially inhibiting cell proliferation (161).

Other methods to facilitate the liquid to gel transition of collagen-based bioink included using temperature-sensitive biomaterials and other natural cross-linkers. Duarte Campos et al. used low gelling temperature agarose as a component in their bovine Col-I based bioink (135). The composition included 2 mg/ml Col-I and 5 mg/ml agarose. The bioink was held in the printer with a temperature above the gelation point and the printed structure was held at room temperature for the gelling of agarose, then at 37°C for gelling the collagen. The structure printed by this bioink can show letters of text placed under it without distortion, and had good cell compatibility (over 95% cell viability); however, it had a lower mechanical strength than cornea (135). Sorkio et al. have used a bioink with 1.2 mg/ml human collagen in combination with human plasma, thrombin, and hyaluronic acid to print the cell-loaded corneal-like structure (145). Thrombin served as a crosslinker to assist bioink gelation. Stem cells were printed by laser-assisted bioprinting in parallel with the main structure, forming a corneal-like structure, with cells surrounded by collagen fibers. The structure showed good cell compatibility; however, it required non-transparent supporting material during printing, resulting in a translucent final structure, with no report of its mechanical properties (145).

A bioink primarily incorporating decellularised cornea was used to print a corneal model using an extrusion printing technique (162). The decellularised cornea was dissolved in acetic acid and pepsin with a concentration of 20 mg/ml and later neutralized by NaOH to make the bioink. The printed corneal model had over 75% light transmittance in the visible light spectrum and was compatible with human turbinate-derived mesenchymal stem cells (hTMSCs) (162). The collagen content of the decellularised cornea solution was suggested to be ~86% (163); however, the detail of the collagen types was not given (163).

In conclusion, the printing method used in published studies is FRESH (158), extrusion printing (160–162), DoD (135), and laser-assisted printing (145). For the crosslinking methods, the chemical crosslinkers that are widely used in fabricating collagen-based corneal-like structures are not popular among the 3-D cell printing projects. As all these projects incorporated cells in the bioink, more gentle crosslinking methods were used that include crosslinking using natural biomaterials, such as alginate-calcium, gelatin and thrombin, and low-temperature agarose. Most of the reported corneal bioprinting research studies used lower concentrations of Col-I (0.82 to 5 mg/ml) to maintain transparency of printed structure but required additional gentle crosslinkers. The only bioink that appears to have a higher Col-I concentration used 20 mg/ml of the decellularised cornea with 86% being collagen (162). This higher concentration of collagen has sufficient printability for extrusion printing without the need to add gelatine (162); however, it would be challenging to define the composition of the material obtained from decellularised corneas that contain many different proteins. This uncertainty could be a significant potential limitation to the broader applicability and use of the bioink. The collagen sources are either animal-based, such as bovine (135, 158, 160) and rat (161), or from human tissue (145, 162). The current studies of 3-D printing of corneal tissue remain primarily proof-of-concept studies that still have a notable period before their actual clinical use. The printed structures are mainly focused on corneal stromal layers and cells, except the study done by Wu et al. that explored the cell viability of encapsulated epithelial cells (161). Although a number of tissues represent potential alternatives to natural tissue, none of the above-mentioned printed structures have reported similar or exceeded the mechanical properties compared to the intact human cornea. It is also notable that none of the above-mentioned projects used photo-crosslinking, either during the printing process or post-printing. This may be because of the concern that the photoinitiator and the light-curing process may be cytotoxic. Sorkio et al. suggested that photocrosslinking can be important not only to further enhance the mechanical properties of the printed structure but also expressed concern for its impact on cell viability (145). Diamantides et al., reported that the cell viability of the chondrocytein, the collagen bioink decreased to 76%, with 10 s of 1.2 W/cm^2^ blue light, and 0.5 mm riboflavin photo-crosslinking (164); however, Ibusuki et al., have shown that with 40 s of photocrosslinking using 0.5 W/cm^2^ blue light and 0.5 mM riboflavin, the cell viability of chondrocytesin, the collagen solution, was still over 90% (81). The cytotoxic effect of photocrosslinking could be reduced by lowering the strength of the curing light, and therefore, it is possible to introduce photocrosslinking into the development of cell encapsulating collagen-based bioinks to enhance methods on bioengineering a cornea.

### Collagen-IV Based Bioink

To date, there is little to no exploration into the field of Col-IV bioprinting, and the utilization of Col-IV as a versatile bioink. Hence, the current use of collagen in bioinks is essentially limited to Col-I as previously discussed. In comparison, the current use of Col-IV emphasizes cell culturing, where it is utilized as a coating. A previous study tested a number of coatings of a polydimethylsiloxane substrate including Col-I and Col-IV (165). Substrates coated with Col-IV were found to produce the most ideal phenotypic expression in bovine corneal endothelial cells, with strong ZO-1 expression and minimal cytoskeletal α-SMA, indicating no abnormal EMT. This was a predicted result as normal corneal endothelial cells have been found to secrete Col-IV as their native collagen, but following an EMT, these cells instead produced Col-I (166, 167). A follow-up study tested cultured primary human cells on coated Col-I gels and found that only gels coated with Col-IV produced confluent monolayers of high cell density suitable for transplant (168). A similar study found that of the different ECM-coating proteins tested, only Col-IV-coated silk fibroin films allowed for the formation of confluent monolayers of primary human corneal endothelial cells that maintained apolygonal morphology (169).

For future applications of bioprinting, including the construction of a full-thickness corneal substitute, Col-IV printing, with or without cells, could hold significant promise. Col-IV bioprinting of layers extends beyond the printing of layers in a potential biomimetic corneal substitute, as this collagen is ubiquitous in the basement membranes of the body. Hence, the development of Col-IV inks and bioinks is essential for the recreation of 3D scaffolds for research, and clinical applications in which the cultured cells require a basement membrane to support their physiological function.

## Conclusion and Future Trends

We have reviewed the application of tissue engineering in the cornea, the lens, and the retina with a focus on Col-I and Col-IV. Compared to the lens and the retina, tissue engineering of corneal structures is heavily studied. This may be due to its relatively simple-layered structure and a strong practical need to overcome the current global shortage of donor corneas. Despite the natural lens representing a relatively simple-structured tissue to the cornea, the success of IOLs has appeared to limit the need for tissue engineering, a lens alternative. Subsequently, most studies found emphasized coating or enhancing the compatibility of IOLs in the lens to reduce the need for secondary cataract (PCO). The more complicated structure of the retina has made it the most challenging to engineer; however, a few studies have successfully engineered retinal-like layers and structures, albeit with variable current practical application (29, 122, 139).

Collagen is a widely used biomaterial and a key structural protein in ocular tissues; however, most studies focused on the application of Col-I. Various methods have been developed to make Col-I- based structures, and all have shared one common principle, that is, to cross-link Col-I fibers. As some of the cross-linking methods, such as gentle cross-linking using alginate, or photo-crosslinking, are compatible with 3D printing, this has enabled further development of printing Col-I based structures. In the cornea, numerous studies have developed various types of Col-I bioinks and printed cell-laden corneal-like structures that have shown similar morphology and transparency to the native intact cornea, albeit with limited comparable tensile strength in many examples. Despite Col-IV being an essential component for lens and basement membranes in the cornea and the retina, it was mainly used as a coating material to support cell growth. Development of a Col-IV based scaffold or bioink remains limited. It could be that fabricating a Col-IV based structure is more challenging than Col-I, given their structural differences, but it can also be that the importance of incorporating Col-IV in tissue engineering for the lens and the retina has not yet been widely investigated. Col-IV is an essential protein for retinal and lens cell growth, and to develop a Col-IV based structure could greatly enhance cell compatibility.

The application of 3D printing was not limited to print an entire structure but also used for *in situ* printing to fill or seal injuries. *In situ* printing is novel in treating diseases, and has been used to treat cartilage injuries (131, 156). With the right biomaterial and biopen developed to fit the size of different ocular tissues, this could provide a useful tool to treat ocular injuries and diseases. Based on the published findings, it is no longer a technical barrier to produce bioengineered ocular tissues, at least not for cornea, in the laboratory; however, translating these developments to the clinic or surgery remains challenging. The complexity of bioengineered tissues, including both biomaterials and cells, and the unique manufacturing process makes these products distinct from other clinical products currently being regulated (170). Their mechanisms of action do not fall into the existing regulatory definition of potency, and long-term survival and integration of bioengineered tissues in host tissues remains unknown and requires ongoing, careful assessment (170). A whole new system that involves regulatory bodies and policymakers is likely required.

## Author Contributions

YS and MO conducted literature search, structured the layout, and wrote the majority part of paper. JF wrote the retina related section and conducted literature search. CH contributed in structuring and reviewing the paper. JY and GS contributed to conception of design and review of the paper. JY also contributed to literature search. FL contributed to the critical analysis of key literatures of the paper and review of the paper. All authors contributed to manuscript revision, read, and approved the submitted version.

## Conflict of Interest

The authors declare that the research was conducted in the absence of any commercial or financial relationships that could be construed as a potential conflict of interest.

## Publisher's Note

All claims expressed in this article are solely those of the authors and do not necessarily represent those of their affiliated organizations, or those of the publisher, the editors and the reviewers. Any product that may be evaluated in this article, or claim that may be made by its manufacturer, is not guaranteed or endorsed by the publisher.
